# The Public Health: Interviews with Local Authorities

**Published:** 1922-08

**Authors:** 


### THE PUBLIC HEALTH.

### INTERVIEWS WITH LOCAL AUTHORITIES.

### II.—THE COUNTY OF DURHAM.

WE have read somewhere that a gentleman from the South some years ago went North to fill a position in a large colliery. On arriving, he was curious to know what was the purpose of the rows of cottages which he saw, and was informed that they were pitmen's houses. "Good gracious!" he exclaimed, "I thought pitmen lived in the pit." We, of course, went into the County of Durham in a much better-informed state of mind. We also knew that, notwithstanding the rows of cottages, Durham, like other parts of the country, has her housing problem. But we had much to learn. We inquired what was the first and foremost need in the county, in the domain of Health. And the answer was, housing. And the second need? Housing. And the third? Housing. The County Health Committee, under the Chairmanship of Alderman Major, kindly placed much interesting information at our disposal, but we shall not attempt within the limits of this article to do more than deal with two or three special features of the County's health activities and requirements. The County is fortunate in possessing so progressive and enlightened a County Medical Officer as Dr. Eustace Hill, who does not leave the Urban and Rural District Councils or the mine-owners any ground for contending that the need for more houses and better houses, and for other sanitary measures, has not been sufficiently brought under their notice.

#### Housing.

If you read that there are some 600,000 acres of land in the Administrative County and a population of about 950,000, and do a simple sum in arithmetic, you find that, on average, there is for every individual about two thirds of an acre of land. It seems quite a nice breathing space. But arithmetic is a sad guide. Read this extract from a report of the Superintendent Health Visitor for the County of Durham, dated April 7, 1922:—

"In several instances where families are living under really dreadful conditions the owners of the property refuse to do any repairs as they require the premises for business purposes, and are anxious to get the tenants out, but the latter cannot get accommodation elsewhere. In one house I personally visited, five families occupied five rooms; the house was old and in very bad repair, many of the windows unopenable owing to old rickety dangerous frames and cords; all the inmates, especially the women and children, looked very sickly."

Dr. Cameron, the Deputy County Medical Officer, is indeed well qualified to speak on the housing conditions and needs in the County, having studied the problem, literally, up hill and down dale. In Dr. Eustace Hill's absence, he informed us as follows:

In March, 1921, he said, we were able to report, after close consultation throughout with the Housing Commissioner for the area and giving him every possible assistance, that housing schemes had been adopted in most sanitary districts of the County, and that in numerous instances houses were being erected with the help of Government grants. A complete survey of the requirements of the County was made, and in a return, which takes very little account of reconstruction needs, we showed, for each of the 29 Urban Sanitary Districts and the 14 Rural Districts, that some 46,000 houses were estimated by the Local Authorities themselves to be needed, of which 42,000 would be built by the Local Authority and 4,000 by private enterprise.

The excess in the general death rate, zymotic death rate, and infant mortality rate in the mining districts of Durham and Northumberland as compared with the rest of the country is almost entirely the result of the unsatisfactory housing conditions which exist.

#### Overcrowding—The "Free House" System.

The Census Commissioners regarded as overcrowded any tenement containing more than two persons per room, and on this basis the overcrowding in the Counties of Northumberland and Durham is three times as high as in the whole of England and Wales, and very greatly in excess of that in other mining districts. The problem was bad enough in the period before the War. In the three years 1912, 1913 and 1914, new houses were erected, but the deficiency grew during those years by a net figure of about 1,000 houses, owing to the concurrent closing and demolition of insanitary dwellings and the growth of population. We could multiply instances of overcrowding, both in the mining and in other areas, from page after page in our reports, but perhaps the following extract is a sufficient illustration of the problem and of our difficulties:—

"In a large proportion of the cases of overcrowding, tubercular patients were in residence. As in the preceding years, practically nothing was done to remedy matters. The County Medical Officer has written scores of letters urging the District Medical Officers of Health to take steps to remedy these gross cases of overcrowding, and the replies invariably indicate the writer's despair of being able to do *anything* until new houses are built. In one large urban district on Tyneside there were 18 reports concerning 28 rooms occupied by 124 people, of whom 22 *were suffering from tuberculosis.* Included there were two cases of one-room tenements with five occupants, and three cases of one-room tenements with six occupants, with a tuberculous inmate in every instance.

. . . .

Obviously it is utterly impossible to deal with the large number of very insanitary areas and properties until there is other available accommodation for the occupants to go to."

The deplorable deficiency in houses in the mining areas, and in the character of the accommodation provided, is mainly a growth of the last 50 years. The "free house" system is the distinctive feature of the Northern Counties mining districts, and is the main cause of this trouble. It is a custom which dates from feudal times, whereby the coal owners provide the married miners with a free house and coals, the invariable arrangement in other parts of the country being that the miner pays a rent for his dwelling. When the free houses commenced to become insufficient for the miners entitled to them, a rent allowance of about 3s. a week was paid. The miner, rather than pay the extra money necessary to rent a house, preferred to put up with overcrowding of the free houses. Again, a miner entitled to a free house is bound to take it or lose his rent allowance. The "free house" system also tends to stifle complaints, because the tenants are afraid that the intervention of the sanitary authorities may lead to their having to take a rented house and pay more rent or even leave the district because another house is not to be had. The miners also lend support to the "free house" system because, during sickness and also during colliery stoppages, they continue to occupy their dwellings rent free. It is only fair to state that, with one or two notable exceptions, the houses erected during recent years by colliery owners in mining districts compare favourably with those built by the speculative builder.

Representatives of the County Council and the local sanitary authorities met the colliery owners in July, 1919, and pressed that (1) they should undertake the repair and renovation of defective houses, (2) rebuild where necessary, (3) abolish all back-to-back houses, (4) procure the proper paving and draining of streets; (5) provide baths wherever possible, and (6) abolish the ash-privy system and substitute water carriage in every district where there are proper sewers and a water supply. The last requirement is one which it would seem almost superfluous to make; but unfortunately there have been actual cases where, in mining districts served with a good water supply and drainage, the insanitary ash-privy was being provided for new dwellings. No definite undertaking was given by the owners, owing to unsettled conditions of the coal industry and for other reasons.

### Stoppage of the Government Grants.

And it is depressing to add that in the absence of further Government grants the local sanitary authorities will not now build, and there is little prospect at present of even 20 per cent. of the houses required being built. When the Minister of Health stands up in Parliament and says that the supposed demand for houses rests to some extent upon the enthusiasm of particular people dealing with particular schemes, and that there is something of a slump in the housing demands, he fails to realise at any rate the position in this part of the country. It does not help us much to be told, as we have been told, that "the housing conditions of the County of Durham will continue to receive the most careful consideration of the Department," when we know that the housing schemes in the county are being held up or very seriously curtailed.

Too wide a publicity cannot be given to this question of the housing necessities of our great northern mining areas. The picture, sketchy and imperfect as it must be within the limits imposed by the space of a short article, is one for the visitor to Durham to reflect upon as an unpleasing contrast to the impression of beauty left on his mind by the majestic cathedral and castle set high up above the banks of the Wear.

### Sanitary Requirements and Water Supplies.

When the miners' houses were erected in rows on the sides of the moor, it is at least a matter for thankfulness that wide spaces were left between the rows, down which the winds may sweep and keep down the pestilence which is too often invited by the insanitary conditions and the unpaved and undrained streets and the ash-privies. The County Council has constantly under review the question of water supply in different parts of the county, and, after conferences with the district sanitary authorities, has obtained Parliamentary powers authorising the formation of a Joint Water Board and the acquisition of the undertaking of the Weardale & Consett Water Company. These powers have been acted upon, and the Joint Water Board has since obtained further powers for the construction of a large impounding reservoir in the western part of the county. We are constantly pressing the substitution of water-carriage for the objectionable ash-privy system, which is all too common. It is disturbing to have to say that where that system persists, it is still too often necessary to bring pressure to bear for such an elementary matter as scavenging to be more efficiently carried out. More attention is now, however, being given by the district authorities to the question of motor haulage for this work. Street-making is crying out for attention in many parts of the county, where mud, almost ankle deep from one side of the street to the other, would come as an unpleasant surprise to those used to granite pavements and macadamised roads.

### The Death Rate.

Let us turn to a pleasanter picture. The death rate has fallen in six quinquennial periods, 1891 to 1920, thus, 19, 18, 17, 16, 15, 14—the last drop notwithstanding the 4,000 deaths from the influenza epidemic in 1918-19, but for which the death rate in the last quinquennial period would have been 13. The mortality from enteric fever, for which at one time the county enjoyed the unenviable reputation of holding the record, shows a progressive and marked decline which is as gratifying as it is difficult to understand.

### Welfare Work—The Value of the Notification of Births Act.

The improvement in the death rate generally, and also in the infant mortality rate, which has fallen from 163 per 1,000 in 1891-1905 to 113 per 1,000 in 1916-1920, is attributable, in our view, mainly to the better education of the people especially in domestic hygiene. Just as we place housing as the paramount need for the county, so we select, without hesitation, the Notification of Births Act, 1907, as the public-health enactment which has been most advantageous in its operation. For it has been the means of bringing those engaged in practical health work into close touch with the people. The excessive infant mortality in the latter part of 1911 brought matters to a head. Letters addressed in that year to the several sanitary authorities with reference to the adoption of the Notification of Births Act brought unsatisfactory replies for the most part, and the Council decided, except in regard to two or three progressive areas, to adopt and administer the Act for the whole county.

Our maternity and child-welfare staff now includes four whole-time women medical officers, four part-time medical officers, a superintendent health visitor, and 68 whole-time health visitors. Our Welfare Centres are well attended—indeed, we have our own overcrowding problem there at times. Our travelling exhibition is constantly moving from place to place, and is a great success. With this exhibition we try to interest our fellow-citizen by showing him the kind of house he ought to live in, domestic contrivances to eliminate expense and unnecessary work, the kind and quantity of food he ought to eat, and we even offer hints as to the proper use of leisure.

A public opinion, so educated and stimulated, is not going to tolerate for all time neglected housing and bad sanitary conditions. The wives of miners and other workpeople by no means resent the visitors' intrusions into their households. They welcome them, and are ready to respond to their suggestions and instructions. There is a bad shortage of trained midwives in the county, but training is rapidly proceeding and the County Council is subsidising the Nursing Association, and also appointing midwives in districts where there is no Nursing Association.

### Ophthalmia Neonatorum.

This is a serious complaint in mining districts, and, realising that blindness ought rarely to result if the case is properly nursed, we spare no effort to ensure that a case reported by a midwife or health visitor is promptly and efficiently treated. Immediately a case is so notified, the general practitioner is wired, or rung up, as to the patient's willingness for the case to be removed, and the ambulance then called up by telephone; sometimes a case reported by telephone 10 miles out in the country has been got into hospital within two hours of the original report.

### The Powers of the County Council.

Enough has been said to show, in regard to sanitary measures especially, that if reasonable efficiency is to be secured and progress made, the Council must, sooner or later, have wider powers, or else local sanitary administration must be made more efficient. In any case the housing problem must receive adequate attention. At present the county council may survey and they may stimulate. This is good, as far as it goes. But they want to see all parts of the county progressing in matters of health and sanitation all along the line, and to have effective powers to that end.

